# Insulin-Like Growth Factor-Binding Proteins of Teleost Fishes

**DOI:** 10.3389/fendo.2018.00080

**Published:** 2018-03-12

**Authors:** Daniel Garcia de la Serrana, Daniel J. Macqueen

**Affiliations:** ^1^School of Biology, Scottish Oceans Institute, University of St Andrews, St Andrews, United Kingdom; ^2^School of Biological Sciences, University of Aberdeen, Aberdeen, United Kingdom

**Keywords:** insulin-like growth factor binding protein, teleost fish, genome duplication, physiology, comparative biology, gene family evolution

## Abstract

The insulin-like growth factor (Igf) binding protein (Igfbp) family has a broad range of physiological functions and a fascinating evolutionary history. This review focuses on the Igfbps of teleost fishes, where genome duplication events have diversified gene repertoire, function, and physiological regulation—with six core Igfbps expanded into a family of over twenty genes in some lineages. In addition to briefly summarizing the current state of knowledge on teleost Igfbp evolution, function, and expression-level regulation, we highlight gaps in our understanding and promising areas for future work.

## Introduction

The insulin-like growth factor (Igf) binding protein (Igfbps) are highly studied, especially in mammals, and a vast literature has emerged on their roles as mediators of Igf signaling events, and diverse functions that extend beyond Igf regulation. This review focuses on the Igfbp family of teleost fishes, which remains poorly understood compared to the mammalian system. Our goal is to summarize the status of knowledge on teleost Igfbps in an evolutionary context, considering work on gene function and physiological regulation, in addition to phylogenetics and genomics. Our coverage of literature is non-encompassing, and we point the reader to additional reviews. The scope of the review is exclusive to the “true” Igfbps, which each bind Igfs with high affinity, rather than the broader proposed superfamily containing Igfbp-related proteins [reviewed in Ref. (1)], that are distantly related in both sequence and function (2). We also assume that the reader has prior knowledge of the core genetic components of the Igf system, where comprehensive reviews with a non-mammalian focus already exist (3–6).

## Origins of the Core Igfbp Subtypes

Gene duplication and subsequent divergence is central to the evolutionary “narrative” of the Igfbp family. While it is long-established that many vertebrates possess six ancestral subtypes (Igfbp-1, -2, -3, -4, -5, and -6), with the primary cDNAs first reported over 25 years ago [e.g., Ref. (7, 8)], their evolutionary origins were elucidated more recently. An important study reported in 2011 (9), built on past work revealing linkage between Igfbp genes and Hox clusters [e.g., Ref. (10); Hox clusters being well-established markers of genome duplication events], to present a realistic scenario for the origin and expansion of core Igfbp subtypes. The hypothesis is that an ancestral Igfbp gene was duplicated in tandem during an early stage of vertebrate evolution to produce a pair of Igfbp genes (9, 10). Subsequently, two genome duplication events in the ancestor to extant vertebrates (11) led one gene to give rise to Igfbp-1, -2, and -4, and the other to Igfbp-3, -5, and -6. A single Igfbp is present in amphioxus, a chordate that did not undergo the same duplications, and this molecule failed to bind Igf-I or Igf-II, indicating that Igf-binding is either a vertebrate-specific function (12), or was secondarily lost. The same study confirmed that Igf-independent functions had evolved before vertebrates (12).

It is also important to remember that the diversification of the core vertebrate Igfbp system occurred alongside expansions in other key gene families within the Igf system, including both hormones (13) and receptors (14). It now seems certain that the early vertebrate genome duplication events were crucial for the evolution of distinct insulin and Igf systems [e.g., Ref. (15)]. For the remainder of this review, we focus on the Igfbp system of teleosts, where additional genetic expansions—some dramatic—have been recently characterized.

## Expansions in the Teleost Igfbp Gene Repertoire

A further round of genome duplication occurred in the ancestor to extant teleost fishes (i.e., around half of known vertebrate species) 300–350 million years ago (11). This led to retention of duplicated copies (paralogs) for all the core Igfbp subtypes barring Igfbp-4, where one paralog was lost early (9, 16). In different lineages that have not experienced further genome duplication events, the number of Igfbps retained is variable, but always higher than mammals and most non-teleosts. For example, zebrafish (*Danio rerio*), the most studied teleost in terms of Igfbp function, retains nine unique genes. This includes paralog pairs for Igfbp-1 (17), -2 (18), -5 (19), and -6 (20), along with a single Igfbp-3 copy and no Igfbp-4 gene, owing to lineage-specific losses (9, 16). The phylogenetic relationships of teleost Igfbp paralogs have been established using robust methods (9, 16). An “-a”/“-b” nomenclature common to different teleosts is preferred (e.g., “Igfbp-1a” and “-1b”) (16), as it acknowledges a common ancestral origin from the same duplication event, while accommodating zebrafish nomenclature [e.g., Ref. (17–20)].

**Figure 1:**
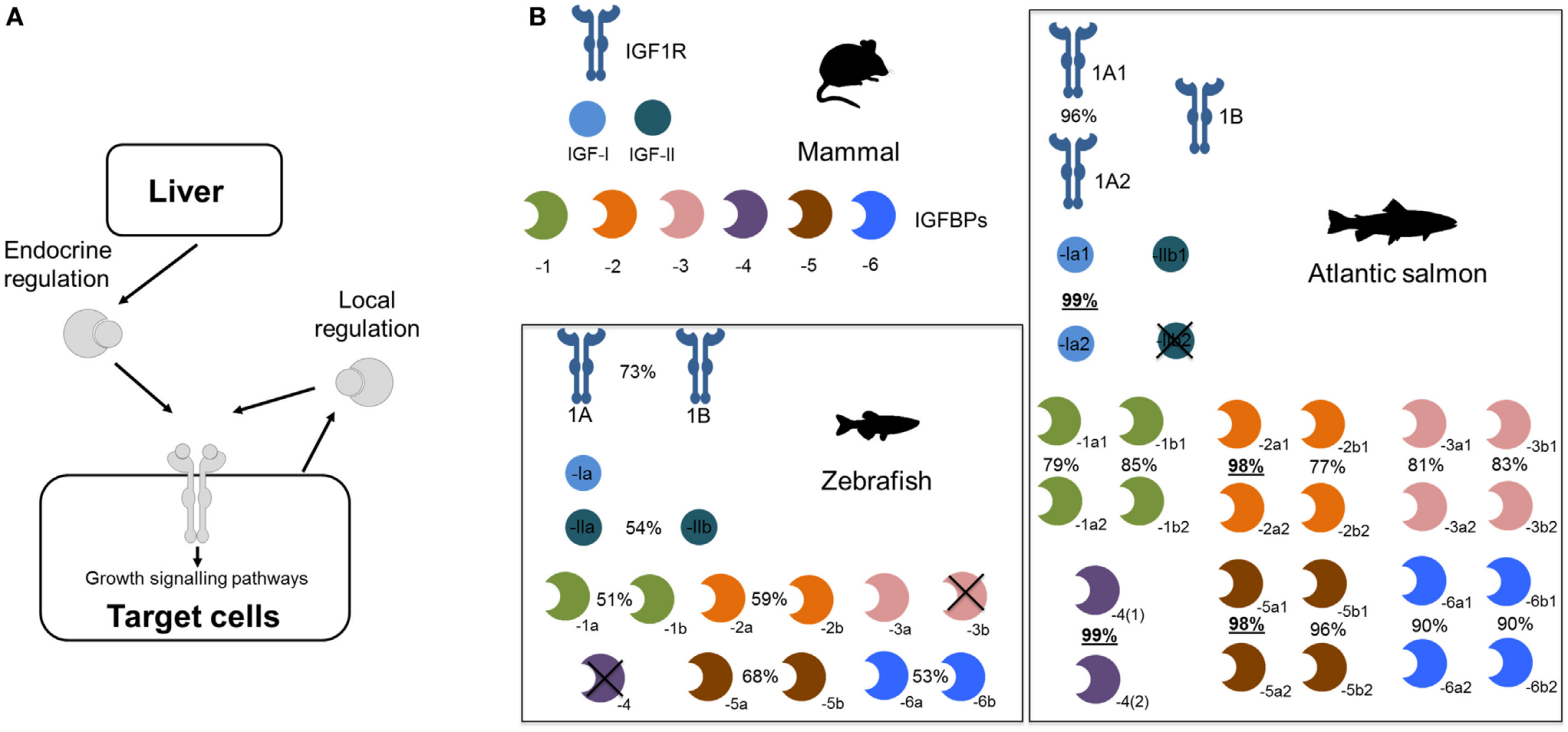
Expansion of the core insulin-like growth factor (Igf) system—including Igfbps—during teleost evolution. **(A)** Simplified depiction of Igf system. **(B)** Core Igfbp system components (i.e., proteins encoded by distinct genes) in different vertebrate groups, contrasting a typical mammalian system with that of two teleost lineages. For teleosts, % identity is shown for paralogous amino acid sequences. For Atlantic salmon, the underlined % identities highlight paralog pairs residing in regions of the genome that experienced a delay in cytological rediploidization after genome duplication (24), a process required for paralogs to diverge in sequence on distinct chromosomes—hence, these genes have had less evolutionary time to diverge, leading to extremely high identity. Phylogenetic relationships of the Igfbp families from these different lineages, along with another group of teleosts that experienced a separate lineage-specific genome duplication event, are depicted in Figure 2.

Several teleost lineages experienced additional rounds of genome duplication. This includes a well-studied event ~95 million years ago in the salmonid ancestor (21, 22) that caused dramatic genetic expansions within the Igf system (summarized in Figure 1). For example, we reported in 2013 that salmonids retain at least 19 unique Igfbp genes, with salmonid-specific paralogs of *igfbp-1a, -1b, -2b, -3a, -3b, -5b, -6a*, and -*6b* (16). We proposed a nomenclature with either “1” or “2” after the “a” and “b” teleost symbols (e.g., “*igfbp-1a1*” and “-*1a2*”). Several of these Igfbp pairs are highly divergent compared to the genome-wide average for paralogs retained from the salmonid genome duplication event (Figure 1; e.g., sharing <80% amino acid identity, compared to an average of ~93% across thousands of paralog pairs) (23). This points to functional divergence at the protein level that remains entirely unexplored.

More recently, an improved understanding of the complexities of genome evolution following the salmonid genome duplication, which was a spontaneous genome doubling event (or “autotetraploidization”) (21, 24), led to the discovery of salmonid Igfbp paralog pairs for *igfbp-4, igfbp-2a*, and *igfbp-5a* (24), which share extremely similar sequences and were previously unrecognized or ignored as alleles (see Figure 1; legend contains additional information). Thus, some salmonid species, including the commercially important Atlantic salmon (*Salmo salar*), possess 22 unique *igfbp* genes, with 11 paralog pairs (Figure 1), some of which may have evolved adaptively (25). Remarkably, every possible Igfbp paralog generated from the salmonid-specific genome duplication was evidently maintained, despite the genome-wide paralog retention rate being around 50% in the same species (21). We and others have also identified expansions to other core gene components of the Igf system due to the salmonid genome duplication, including Igf-I [e.g., Ref. (24, 26)], Igf-II (25), and Igf-1R (27). These paralogs remain of substantial interest, but we are at an early stage of understanding their roles in salmonid biology.

**Figure 2:**
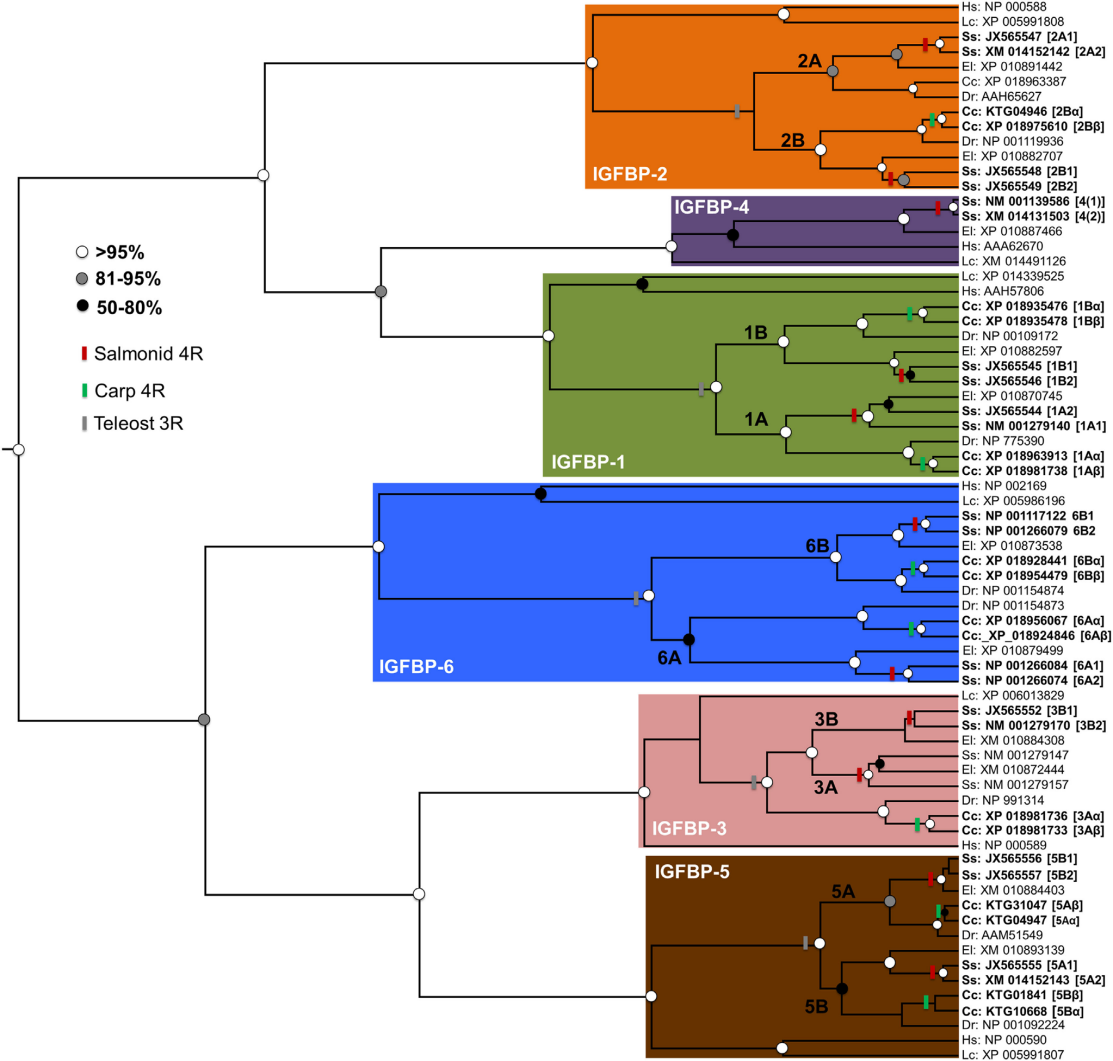
Independent evolutionary expansions to the Igfbp family of teleosts. A phylogenetic analysis was performed, including 71 unique Igfbp amino acid sequences from a standardized set of taxa: Human *Homo sapiens* (“Hs”), coelacanth *Latimeria chalumnae* (“Lc”), zebrafish *Danio rerio* (“Dr’”), common carp *Cyprinus carpio* (“Cc”), Atlantic salmon *Salmo salar* (“Ss”), and northern pike *Esox lucius* (“El,” from a sister lineage to salmonids that did not undergo the salmonid-specific genome duplication). Accession numbers are given for all sequences, which were gathered from the NCBI RefSeq database, facilitated by BLAST analyses (32). The sequences were aligned using Mafft V.7 (33) with default settings. Alignment quality filtering was done using the Guidance2 algorithm (34) to remove the least confidently aligned regions. This led to a high-confidence alignment of 212 amino acids positions (SI file 1). The alignment was used in Bayesian phylogenetic analysis, using methods published elsewhere (35). Briefly, this was done in BEAST v. 1.8 (36) using the best-fitting amino acid substitution model (JTT+G+I), determined by maximum likelihood *via* the IQ-tree server (37), along with a relaxed molecular clock model (38), allowing probabilistic estimation of the trees root [consistent with previous studies (9, 16)]. The tree is annotated to show genome duplication events in teleost evolutionary history, including in the teleost ancestor (“3R”) and additional events in the salmonid and carp lineages. The nomenclature for salmonid and carp paralogs is given as described in the text. Branch support values (posterior probability) are highlighted by circles placed on nodes, with colors matching a legend. Minor inconsistencies in branching patterns in some Igfbp clades (e.g., relating to the 3R or salmonid 4R) compared to other studies (16), can be explained by the short alignment length. Nonetheless, the tree demonstrates independent expansions within the salmonid and carp Igfbp repertoire, additional to paralogs retained in many other teleosts.

Additional lineage-specific genome duplication events have occurred in several teleost groups, including the ancestor to goldfish (*Carassius auratus*) and common carp (*Cyprinus carpio*). This event is younger than the salmonid-specific genome duplication event, occurring around 8–12 million years ago (28, 29). It also involved a distinct mechanism (“allotetraploidization”), where two species hybridized before genome duplication (28). This event created a large set of paralogs (28), some of which are known to have experienced functional divergence [e.g., Ref. (29, 30)]. However, no accompanying expansions to Igfbp repertoire are yet reported. To explore this knowledge gap, we performed a bioinformatic and phylogenetic analysis, revealing that common carp retains at least 17 unique *igfbp* genes, including paralog pairs for *igfbp-1a, -1b, -2b, -3a, -5a, -5b*, -*6a*, and -*6b* (see Figure 2; methods provided therein). To avoid confusion with the salmonid-specific paralogs, we suggest “α”/“β” is added to the existing teleost nomenclature when these duplicates are studied in the future (e.g., *igfbp-1a*α/*-1a*β) (Figure 2). The results confirm that salmonids are not unique among teleosts in retaining a highly expanded Igfbp repertoire. In fact, as many vertebrate groups have experienced lineage-specific genome duplication events, both fishes and non-mammalian groups, including anuran frogs [e.g., Ref. (31)], it seems likely that many other species possess expanded Igfbp repertoires, contributing additional complexity to their growth regulation.

## Physiological Roles of Teleost Igfbps

Many studies have investigated the physiological roles of Igfbp genes in the teleost lineage. Barring a few model species (e.g., zebrafish), it has been historically challenging to perform functional analyses in most teleosts, although this is changing in light of emerging genome-editing methods (see Perspectives and Future Work). Hence, while in mammals, Igf-dependent and Igf-independent functions have been widely demonstrated, the majority of studies in teleosts have failed to reach similar levels of functional insight. In fact, most work has focused on expression-level regulation of *igfbp* genes or proteins under a diverse set of experimental stimuli. It is also important to note that most teleost Igfbp research has focused on aquaculture species of high commercial value, including the salmonid, perciform (perch-like fish), pleuronectiform (flatfish), cypriniform (carp spp. and relatives), and siluriform (catfish) groups. This has led to a bias toward physiological processes relevant to commercial production, especially growth, muscle development, stress, and disease resistance. In this section, we briefly summarize the literature on teleost Igfbp function and regulation, considering the core vertebrate subtypes separately. We make attempts to distinguish Igfbp paralogs according to the evolutionary histories and nomenclature described above, although this is often not possible as many studies failed to distinguish paralogs, especially for the most recently discovered genes.

### Igfbp-1: A Negative Regulator of Teleost Growth

In mammals, Igfbp-1 is mainly produced in the liver and secreted to circulation, where it acts to limit Igf signaling in catabolic contexts, such as fasting, stress, and hypoxia (39). It is widely considered a negative regulator of somatic growth, reproduction, and development (4, 40); and interacts with cell surface integrins to stimulate cellular motility (41). It has Igf-dependent and Igf-independent functions, along with important roles in the regulation of metabolism [reviewed in Ref. (42)].

In salmonids, Igfbp-1a and Igfbp-1b are two of the three major circulatory Igfbps (43), first identified by molecular weight (20–15 and 28–32 kDa, respectively) (4, 44). It is likely that similar molecular weight Igfbps detected in others teleosts plasma are Igfbp-1 orthologs (45–49). Igfbp-1 encoding genes, as in mammals, are mainly expressed in teleost liver (16, 50–55). In zebrafish embryos, *igfbp-1a* mRNA is expressed during early development; while *igfbp-1b* is expressed later, after which time both paralogs become restricted to liver (17, 50). At the functional level, both Igfbp-1a and -1b of zebrafish can bind to Igf-I and Igf-II, but Igfbp-1b had a lower affinity for each hormone, and a lesser ability to downregulate Igf-I signaling (17). In other species, it has been reported that *igfbp-1a* genes are expressed in non-hepatic tissues, but typically at lower levels than in liver (16, 51, 52, 54–58). This supports the hypothesis that Igfbp-1a evolved more localized functions than Igfbp-1b (6).

Several teleost studies have reported protein or transcript level upregulation of Igfbp-1 genes during catabolic a process, which probably serves to downregulate growth by sequestering Igfs from Igf-1Rs, allowing allocation of resources to metabolic processes essential for survival. Consistent with these findings, overexpression of *igfbp-1a* (and *igfbp-1a* in zebrafish) in cypriniform embryos (17, 54, 59) caused growth and developmental retardation. Nutrient deprivation has been shown to increase circulatory Igfbp-1 proteins and *igfbp-1* gene expression in liver (for both teleost paralogs, when distinguished) and skeletal muscle *igfbp-1a* expression, which is reversed by a return to anabolic conditions (50, 51, 55, 60–65). It has also been shown that dietary amino acid deficiency can upregulate *igfbp-1* gene expression through a not well-described mechanism (64). Teleost *igfbp-1* genes are also negatively regulated by growth hormone (Gh) and Igf-I, consistent with a negative role in growth regulation (44, 46, 48, 53, 57, 62).

*Igfbp-1* expression in teleosts is also modulated by hormones others than the Igfs. For instance, *igfbp-1a* evidently plays a role in zebrafish sexual maturation, in a way that seems to differ from its classical anti-proliferative role. Specifically, *igfbp-1a* expression increases in response to T_3_ and the follicle stimulating (Fsh) hormones (well-known for stimulating spermatogonia proliferation) (58). The female sex hormone 17β-estradiol also increased Igfbp-1 secretion in striped bass liver explants (46).

Stress is also known to induce *igfbp-1* gene expression in teleosts. Cortisol, the main stress hormone in vertebrates (66, 67), and other synthetic glucocorticoids such as dexamethasone increases both Igfbp-1a and Igfbp-1b circulatory levels, as well as gene expression in liver and cultured myotubes (43, 57, 62, 68, 69). Environmental stressors, such as hypoxia, confinement, temperature, heavy metals, and salinity, were also found to increase *igfbp-1* expression (17, 49–53, 56, 61, 70, 71). It is also possible that *igfbp-1* upregulation in response to food deprivation (see above) is related to a rise in cortisol, as some studies have found increasing levels of circulatory glucocorticoids in response to fasting (70, 72, 73). There is also emerging evidence from salmonids that *igfbp-1a1* upregulation during infection serves a role in linking growth to innate immunity, potentially promoting downregulation of growth in favor of a more effective immune response (74). This expression response represents an example of salmonid-specific divergence in the regulation of Igfbp paralogs, as *igfbp-1a2* was unaltered by infection in the same study (74).

### Igfbp-2: A Major Circulatory Igfbp in Teleosts

In mammals, Igfbp-2 is highly expressed during embryonic stages, and more lowly expressed in adult tissues, with highest levels in liver, adipocytes, the central nervous and reproductive systems, heart, and kidney (75). Mice embryos overexpressing *Igfbp-2* show a reduced growth rate, likely through reduced Igf availability (76). However, Igfbp-2 deletion in mice embryos does not have any significant effect on growth or development (77), which may indicate compensatory effects with other Igfbps. While the functional roles of Igfbp-2 remain relatively poorly established, recent studies have linked it to several pathological states. For example, *Igfbp-2* may act as a tumor promoter (78) by suppressing epidermis growth factor receptor nuclear signaling (79). There is also increasing evidence that Igfbp-2 plays a role in mammalian bone formation (80).

Igfbp-2b is the third main circulatory Igfbp in salmonids and probably other teleost species (41 kDa form) and the main Igf carrier (6, 81, 82). For a long time, Igfbp-2b was wrongly considered to be Igfbp-3 (83) due to its similar physiological regulation to Igfbp-3 in mammals [e.g., Ref. (83)]. Teleost *igfbp-2* genes are expressed in a range of tissues [e.g., Ref. (16, 84–86)] with zebrafish *igfbp-2a* and *igfbp*-*2b* having different spatiotemporal patterns during early development, and each being expressed in liver in adults (18). In adult salmon, *igfbp-2a* was expressed across multiple tissues, with especially high abundance in liver, whereas *igfbp-2b1* and *igfbp-2b1* were liver-restricted (16). Overexpression of *igfbp-2a* and *igfbp-2b* causes a reduction in growth and developmental rate in early-stage zebrafish (18, 87), suggesting an equivalent role to that observed in mammals. Similar to Igfbp-1, past work has suggested a role for Igfbp-2 in teleost sexual maturation, with *igfbp-2* mRNA being expressed in pre-ovulatory ovaries of rainbow trout, and upregulated in response to female sex hormones (83).

Mixed results exist on the regulation of teleost *igfbp-2* genes by nutritional status. For example, some past studies showed that *igfbp-2* genes are downregulated or unchanged in liver and skeletal muscle of fasted fish (55, 88–90), which does not support an obvious role in growth inhibition. By contrast, a significant increase of *igfbp-2* expression was observed in fasted zebrafish (91), although this same effect was not clearly observed in a later study of zebrafish that distinguished *igfbp-2a* and *-2b* (65). In Atlantic salmon, a significant decrease in *igfbp-2a* (formerly “*igfbp-2.1*”) expression was reported in skeletal muscle during refeeding after a period of restricted food intake, suggesting an inhibitory role on growth (90, 92). Similarly, all three tested *igfbp-2* paralogs (*igfbp-2a*, -*2b1*, and -*2b1*) were downregulated in Atlantic salmon liver upon post-fasting refeeding, again suggesting an inhibitory role on growth (16). However, such data have not been replicated *in vitro* where neither amino acid deprivation nor addition of Igf-I and amino acids to Atlantic salmon cultured myotubes modified the expression of the same paralogs (57).

Moreover, the regulation of *igfbp-2* expression by Gh does not clearly support a growth inhibitory role common to teleosts. While a study in zebrafish embryos reported that Gh inhibits *igfbp-2* expression (91), work in Atlantic salmon demonstrated an increase in circulating Igfbp-2b in response to Gh (48, 81, 93). By contrast, treatment with dexamethasone, known to enhance catabolism, led to an increase in *igfbp-2a* expression in salmon skeletal muscle myotubes (57). Despite not distinguishing teleost paralogs, recent work revealed upregulation of skeletal muscle *igfbp-2* expression in fine flounder (*Paralichthys adspersus*), concomitant to a rise in blood cortisol (94). Differences in Igfbp-2 expression across studies suggest a complex role for this Igfbp family member in teleost growth, dependent on both physiological and species context.

### Igfbp-3: Divergent Physiological Regulation across Teleost Species

Igfbp-3 is the main carrier of circulating Igf in mammals, forming a tertiary structure with the acid-labile subunit (ALS) that increases Igf half-life and regulates Igf bioavailability (95). Igfbp-3 has anti-proliferative effects in many mammalian cell types, preventing the interaction of Igf-I and Igf-II with Igf-1R, and it also has Igf-independent roles (96). In this respect, Igfbp-3 directly interacts with two-cell surface receptors independently of Igf-I, Lrp1, and Tmem29, which mediates its anti-proliferative effects (97, 98). However, mammalian Igfbp-3 can also enhance cellular proliferation in some conditions, through both Igf-dependent and Igf-independent mechanisms (99, 100).

In contrast to mammals, teleost Igfbp-3 proteins are not considered major circulatory Igfbps (6). In fact, there exists no known association between Igfbp-3 proteins—or indeed any teleost Igfbp subtype—and ALS (6), highlighting fundamental differences in the way Igfs are regulated in circulation. In zebrafish, the single *igfbp-3a* (16) gene has important roles in early development, showing dorsalizing effects in embryos through an Igf-independent interaction with bone morphogenic protein 2b (101), one of few studies demonstrating an Igf-independent role for a teleost Igfbp. The four distinct *igfbp-3* paralogs of salmonids (*igfbp-3a1*, -*3a2*, -*3b1*, and -*3b2*) were lowly expressed in 11 tested adult Atlantic salmon tissues (and each absent in liver), although *igfbp-3a1* was among the most abundant of all Igfbp family member genes in heart (16) and the only detected *igfbp-3* gene in primary myotube culture (57). Conversely, *igfbp-3b* of adult fine flounder was reported as more highly expressed in liver (the main route for Igfbp to circulation) than several other tested tissues, while *igfbp-3a* was not considered in the same study (88).

Studies in zebrafish, flounder, and yellowtail reported a significant increase in the expression of *igfbp-3* genes in liver and/or muscle in response to fasting (55, 88, 102), which may act to restrict Igf signaling. However, on the other hand, studies in salmonids have reported no changes in muscle *igfbp-3* gene expression in response to food deprivation (57, 89) with an increase in *igfbp-3a1* expression in liver during post-fasting refeeding, more consistent with a growth-promoting function (16). Similarly, in coho salmon (*Oncorhynchus kisutch*), *igfbp-3a1* muscle expression was increased by Gh transgenesis (103), again supporting a growth-promoting role. However, stress caused a downregulation of *igfbp-3b* gene expression in skeletal muscle of fine flounder (94), which is inconsistent with a role in growth inhibition.

Overall, there is a relatively limited body of research on teleost Igfbp-3 genes, leaving their roles unclear in many species, with the available evidence suggesting functional divergence among different lineages.

### Igfbp-4: Growth-Promoting Role in Some Teleosts?

In mammals, Igfbp-4 is expressed in many cell types and tissues, where it is often considered to inhibit Igf action (104, 105). However, it is also considered to have growth-promoting roles during early embryogenesis, where it enhances Igf-II activity (106). Some studies have reported Igf-independent actions for Igfbp-4, including in relation to the inhibition of apoptosis (104, 105) and cardiogenesis (107).

In teleosts, *igfbp-4* was expressed in most tissues for each species investigated, including Atlantic salmon (16), tiger pufferfish (*Takifugu rubripes*) (108) and fine flounder (88). Moreover, in Atlantic salmon, *igfbp-4* was described as showing high abundance in gill (108). Atlantic salmon was recently shown to retain two highly similar Igfbp-4 paralogs (see Figures 1 and 2), which show conserved regulation across tissues (24). In tiger pufferfish, fasting caused upregulation of *igfbp-4* expression in several tissues, consistent with an inhibitory role on growth (108). In addition, the expression of recombinant pufferfish Igfbp-4 in zebrafish embryos resulted in significant growth retardation (108). However, these findings contrast studies of several species (including salmonids and fine flounder), where *igfbp-4* expression in response to nutritional status manipulation suggested a growth-promoting role. Such work revealed no change in *igfbp-4* expression during fasting (57, 65, 88–90, 92), but upregulation in response to subsequent refeeding (57, 65, 88–90, 92, 109–111). A study of Arctic charr (*Salvelinus alpinus*) showed that dwarf populations with highly restricted growth had lower constitutive *igfbp-4* expression in muscle than populations reaching larger body size (112). A growth-promoting role for *igfbp-4* in salmonids was also supported by a strong positive correlation between *igfbp-4* and several pro-myogenic gene markers during *in vitro* myogenesis in Atlantic salmon (110). Studies of Igfbp-4 expression in response to stress also suggest a growth-promoting role. For instance, addition of dexamethasone to Atlantic salmon myotubes (57), and stress confinement in fine flounder (94) induced a significant reduction in *igfbp-4* expression. Conversely, an increase in *igfbp-4* expression was reported in skeletal muscle during maturation-induced atrophy in rainbow trout (113).

Overall, the available evidence suggests that the physiological role of Igfbp-4, when conserved, differs across species and physiological contexts, though for some lineages, particularly salmonids, a growth-promoting function is implicated.

### Igfbp-5: Conserved Roles in Muscle Growth

Igfbp-5 is the most conserved Igfbp family member. In mammals, it forms a ternary complex with ALS, similar to Igfbp-3, although much more circulating Igf is carried by Igfbp-3-ALS (114). Igfbp-5 represents an essential regulator of many processes in mammalian bone, kidney, mammary gland, and skeletal muscle (114) and can assert both stimulatory and inhibitory effects (depending on cell type) through Igf-dependent or Igf-independent routes. For instance, it has growth factor-like actions, stimulating bone growth in Igf-I knockout mice (115), and smooth muscle cell migration (116). There is also evidence that Igfbp-5 can translocate into the nucleus (117) and have nuclear functions (118). It is thought that Igfbp-5 cellular internalization is achieved by interaction with membrane proteins such as heparin sulfate proteoglycans, and that the Igfbp-5 N-terminal region has an Igf-independent transcriptional activity (118). Furthermore, Igfbp-5 can interact with transcription co-activators such as the four and half Lim domains 2 (119).

In zebrafish and grass carp embryos, *igfbp-5a* and *igfbp-5b* have distinctive patterns of expression during early development, suggesting evolutionary divergence in regulation (19, 120), which has also been demonstrated at the functional level (19). In adult zebrafish, *igfbp-5a* was expressed at high levels in brain and gill, and lower levels in several other tissues, but was absent in liver or skeletal muscle; while *igfbp-5b* was ubiquitously expressed. Similarly, in other studied teleost species *igfbp-5* genes were reported to show a broad tissue distribution, with differences noted among species and paralogs (51, 52, 55, 63, 120), including three paralogs in Atlantic salmon (16, 24).

The importance of *igfbp-5* genes for muscle differentiation and growth in teleosts is well established. *Igfbp-5* expression has been studied across *in vitro* myogenesis, with both teleost paralogs (when distinguished) detected from early stages (i.e., myogenic progenitor cells) through to fully differentiated myotubes (57, 110, 111). In Atlantic salmon, both *igfbp-5a* (formerly: *igfbp5.1*) and *igfbp-5b* (formerly: *igfbp5.2*) showed highest expression in early-stage myoblasts, which decreased during myogenic differentiation (110). Using the same *in vitro* models, it was observed that pro-growth stimuli such as amino acids and Igfs increase *igfbp-5* gene expression (111, 113), including both *igfbp-5a* and *igfbp-5b* paralogs distinguished in salmonids (57, 110). However, *igfbp-5* paralogs appear to have different patterns of expression in response to catabolic signals. For instance, while amino acid deprivation had no effect on the regulation of any *igfbp-5* paralog in Atlantic salmon myotubes (57, 113), dexamethasone reduced *igfbp-5a* expression, while simultaneously increasing *igfbp-5b1* (57). A past study of rainbow trout skeletal muscle recorded no change in *igfbp-5* gene expression in response to fasting or re-feeding (89), though it was unclear which paralog was measured. Similarly, *igfbp-5a* and *igfbp-5b* muscle expression did not change in response to short- or long-term fasting in Atlantic salmon (90, 92). However, in grass carp, *igfbp-5a* and *igfbp-5b* expression decreased in skeletal muscle during fasting, while both paralogs were upregulated in liver, and upon injection of Gh in both tissues (120). In Gh transgenic coho salmon, *igfbp-5b1* was significantly upregulated (103).

There is also emerging evidence that *igfbp-5* genes play a role in ionic homeostasis and Igf regulation in zebrafish (121), stickleback [e.g., Ref. (122)] and Atlantic salmon gills (123) with Igfbp-5a acting to regulate calcium influx in zebrafish gills (121) and being differentially expressed and under divergent selective pressures in marine vs. freshwater sticklebacks (122, 124).

To sum up, the available evidence suggests that Igfbp-5 genes play conserved functions in multiple aspects of teleost biology, with roles most clearly demonstrated in myogenesis, muscle growth, and gill function. There is also considerable evidence that both teleost and salmonid-specific Igfbp-5 paralogs have evolved divergent roles.

### Igfbp-6: A Growth Inhibitor with Emerging Roles

Igfbp-6 represents a special case among the Igfbp family. In mammals, it has a 50-fold binding preference for Igf-II over Igf-I (125, 126) (a unique feature among Igfbps), but also shows differences in key protein motifs, with three disulfide bonds in the N-terminal region instead of the 4 found in Igfbp-1 to 5 (127). Igfbp-6 is a relatively specific inhibitor of Igf-II actions and, therefore, regulates processes where Igf-II is involved such as proliferation, survival, migration, and differentiation (125, 126). Igfbp-6 also has known Igf-independent actions (125, 128), including the inhibition of fibroblast proliferation (129), cancer cell migration (130), and apoptosis (131). The gene has a broad tissue expression distribution in mammals, including lung, liver, and the gastrointestinal tract.

While differences in the affinity of Igfbp-6 proteins for Igf-II and Igf-I are yet to be confirmed in teleosts, the main underlying structural features are conserved (16). In zebrafish adults, *igfbp-6a* was highly expressed in muscle, and almost undetectable in other tissues, while *igfbp-6b* was only abundant in brain, heart, and muscle (20). In adult fine flounder, *igfbp-6b* was most highly expressed in heart, gills, and the gastrointestinal tract (88). In adult Atlantic salmon, neither *igfbp-6a1* nor -*6a2* were notably expressed across a panel of 11 tissues, while *igfbp-6b1* and *6b2* were each broadly expressed, with the latter being especially highly expressed in gill, brain, and spleen (16). Both *igfbp-6b1* and *6b2* were also reported as being highly expressed in Atlantic salmon gills, where they were dynamically regulated during smoltification (123).

The overexpression of either zebrafish *igfbp-6* paralog caused a significant reduction of embryonic growth (20), suggesting a role in growth inhibition. Studies of *igfbp-6* gene regulation in skeletal muscle support a similar role in other species, though some conflicting data exist. For example, a study in Atlantic salmon reported no change in *igfbp-6b* expression in response to food intake manipulation (92), while another reported downregulation of *igfbp-6b* in tilapia skeletal muscle in response to feeding after a period of fasting (53). Similar results were observed in fine flounder skeletal muscle, where *igfbp-6b* expression decreased in response to feeding immediately post-fasting, although expression then increased during long-term refeeding (88). However, *igfbp-6b* was repressed in fine flounder skeletal muscle in response to stress (94), which is less consistent with a negative role in growth. Conversely, in Atlantic salmon myotubes treated with dexamethasone, *igfbp-6a1* was downregulated, while *igfbp-6b2* was upregulated, highlighting complex functions that cannot be easily interpreted without functional data (57).

Recent work also implies a novel role for *igfbp*-6 genes in linking growth and immune regulation in teleosts. Alzaid et al. observed a significant increase of *igfbp-6a2* in primary immune tissues of rainbow trout following a bacterial infection, and provided evidence that immune-responsive *igfbp-6a2* upregulation was stimulated by immune signaling pathways driven by pro-inflammatory cytokines (27). Past work in salmonids has also shown that pro-inflammatory cytokines can promote the expression of *igfbp-6* genes in skeletal muscle cell cultures (132) and *in vivo* (103), which may be linked to the balancing of energetic allocation toward effective immune function.

In summary, Igfbp-6 genes of teleosts are rather understudied, and it is difficult to draw overarching conclusions about their roles and functions at this time.

## Perspectives and Future Work

Our current understanding of the Igfbp repertoire of different teleosts has benefited greatly from recent expansions to genomic resources. We can now be confident in the existence of many teleost paralogs, which are expressed and presumably functional. However, our understanding of the functions and regulatory control of these genes remains highly fragmented across teleosts as a group and remains highly underdeveloped compared to mammals. It is becoming increasingly clear—perhaps with the exception of Igfbp-1—that teleost and mammalian Igfbp orthologs have evolved distinct expression-level regulation. This points to distinct functional roles in the regulation of growth in teleosts compared to mammals, which may be related to differences in growth dynamics, for example, indeterminate growth in teleosts. Moreover, there is also evidence that Igfbp orthologs from different species have evolved distinct regulation and hence, potentially functions, during teleost evolution. This can be speculatively linked to the additional functional flexibility or redundancy linked to Igfbp duplication events, which has allowed divergent regulation of paralogs to evolve under different physiological contexts.

It is also clear that differences in the expression of homologous Igfbp genes across teleost species are often difficult to interpret. In many cases, this may be linked to the historic ignorance of paralogous genes, either by considering one paralog in pair, or detecting signals from both paralogs in gene expression analyses. Hence, a fuller understanding of Igfbp genes will be possible in the presence of high-quality reference genomes, where all genes are properly annotated and can then be distinguished experimentally. The evidence for divergent regulation of Igfbp paralog expression is overwhelming, even for genes with very similar coding sequences (24), suggesting gene expression studies should make every effort to distinguish Igfbp paralogs.

An additional priority for future research should be to characterize the individual protein-level functions of all teleost Igfbp paralogs in multiple species extending beyond model organisms. While it has classically been challenging to perform functional analyses in non-model teleosts, the research landscape is rapidly changing. For example, genome editing using engineered CRIPSR/Cas9 systems has been demonstrated *in vivo* for various large commercial species, including salmonids (133) and catfishes (134), as well as in teleost cell culture (135). Hence, while even 5 years ago, the full repertoire of Igfbp genes was not even recognized in many teleosts, we can look forward to a future where every paralog within a species has its function cataloged by such approaches, even in lineages with hugely expanded Igfbp gene families. This will open the door for associating protein-level functional divergence in Igfbp paralogs with evolutionary changes in gene expression regulation, generating a fuller picture of the biological roles of this fascinating gene family in teleosts.

## Author Contributions

DGS and DJM wrote the manuscript and prepared the figures. DM built the phylogenetic tree showed in the manuscript.

## Conflict of Interest Statement

The authors declare that the research was conducted in the absence of any commercial or financial relationships that could be construed as a potential conflict of interest.
